# The attitudes of fertile and infertile women to Oocyte donation in a Muslim and Secular population

**DOI:** 10.12669/pjms.335.13556

**Published:** 2017

**Authors:** Mehmet Musa Aslan, Vedat Ugurel, Koray Elter

**Affiliations:** 1 Mehmet Musa Aslan, MD. Instructor, Department of Obstetrics and Gynecology, Division of Reproductive Endocrinology and Infertility, Assisted Reproductive Technology Unit, Trakya University Hospital, Edirne, Turkey; 2 Vedat Ugurel, MD. Instructor, Department of Obstetrics and Gynecology, Division of Reproductive Endocrinology and Infertility, Assisted Reproductive Technology Unit, Trakya University Hospital, Edirne, Turkey; 3 Koray Elter, MD. Associate Professor, Department of Obstetrics and Gynecology, Division of Reproductive Endocrinology and Infertility, Assisted Reproductive Technology Unit, Trakya University Hospital, Edirne, Turkey

**Keywords:** Infertility, Oocyte donation

## Abstract

**Objective:**

To determine general attitudes of fertile and infertile women to oocyte donation in a Muslim and secular population.

**Methods:**

The participants consisted of fertile women (n=133) who had at least one healthy living child spontaneously conceived without any fertility treatment and infertile women (n=133) who were diagnosed with primary infertility. Both groups were evaluated with charts comprised of 34 questions addressing demographic characteristics and the social aspects of oocyte and sperm donation.

**Results:**

Although the age of fertile women was significantly greater than infertile women, there was no significant difference in terms of duration of marriage, education level, or employment status between the two groups. Most of the women in each group reported that they did not have enough knowledge about oocyte donation to make a decision. Only 12% of fertile women and 18% of infertile women declared that they would have oocytes from another woman if they did not have or could not have a child (p=0.004). Only 9.0% of fertile women and 18.8% of infertile women declared that they would donate oocytes to anyone who is infertile (p=0.021).

**Conclusion:**

Despite improvement in health care, most fertile and infertile women are still against oocyte donation. This situation may be related to the conservative leanings of Turkish society in recent decades.

## INTRODUCTION

Infertility is defined as the inability to achieve pregnancy despite the fact that couples of reproductive age perform sexually intercourse three or four times a week during the course of one year.^1^-^4^ In Islamic societies, procreation is an important purpose for marriage and it is deemed very important for the stability and happiness of the marriage. For this reason, infertility causes biological, psychological, psychosocial, and cultural problems in infertile couples.

Currently, there are many assisted reproduction models available; some of them involve a third party, such as gamete or embryo donation or surrogate motherhood. Women with ovarian failure were considered irreversibly sterile until about 20 years ago, but this opinion has changed thanks to the improvements in assisted reproduction techniques (ARTs). Today, for women with premature ovarian failure or low ovarian reserve, the chance of pregnancy is recognized as realistic thanks to oocyte donation. Oocyte donation is generally effectuated through in vitro fertilization (IVF) by transferring the oocyte from a healthy young donor after ovarian hyperstimulation and the sperm from the partner of the recipient in the recipient’s uterus. The first successful pregnancy in a recipient woman using donated oocytes was performed in 1983.^5^

Since then, oocyte donation has become a developing area of ART. The purpose of this study was to assess the knowledge levels and approaches of the fertile and infertile families concerning the oocyte donation in a Muslim country.

## METHODS

Our study was performed in the Gynecology Policlinic and ART Center affiliated with the Department of Obstetrics and Gynecology of Trakya University Faculty of Medicine. The institutional ethics committee’s approval was obtained. In the study, 133 fertile and 133 infertile (and non-menopausal) women aged between 18 and 45 years old were involved. The fertile group (Group-1, n=133) was randomly assigned from women who had at least one healthy living child conceived spontaneously without any fertility treatment; the infertile group (Group-2 n =133) was comprised of patients who were diagnosed with primary infertility in the ART unit between January 2015 and December 2015. The women participating in the study provided their informed consent.

Statistical analysis was performed using the SPSS for Windows software, version 22 (Chicago, Illinois). The Kolmogorov–Smirnov and Shapiro–Wilk tests were applied to the variables corresponding to a normal distribution. Continuous variables are expressed as mean + standard deviation, whereas categorical variables are displayed as numbers and percentages. Student t-test and nonparametric Mann–Whitney U-test were used to determine the differences between mean values for normally and non-normally distributed variables, respectively. Categorical variables were reported as percentages and were analyzed by either the chi-square or the Fisher exact test, as appropriate. The statistical significance level was accepted as p≤0.05.

**Table-I:** Questions about demographic data and oocyte donation.

	*Age*	
Demographic Data	For how many years are you married?	
	Are you employed?	Yes
		No
	Your monthly income	Low
		Normal
		High
	Your education level	Illiterate
		Primary
		High School
		University
	Do you have knowledge about oocyte donation?	Yes
Oocyte donation		No
	Would you accept oocytes from donors if you had not or could not have a child?	Yes
		No
	Would your husband think of having a child from oocyte donation if you had not or could not have a child?	He would accept
		He would not accept
		I do not know
	Would you donate oocytes to someone else who wants to have a child from oocyte donation?	Yes
		No
	Would you accept oocytes from one of your close relatives?	Yes
		No
	Would you donate your oocytes to one of your close relatives?	Yes
		No

## RESULTS

The demographic data of participants (age, education level, marriage duration, employment status) is presented in Table-II. The age of fertile women was significantly higher than that of infertile women. Marriage duration, education level, and employment status did not differ between the groups. Most of the women in both groups had graduated from university. Most of the women in both groups were employed. Infertile women had a significantly higher ratio of ’high income’ with respect to fertile women. The distribution of answers to questions about oocyte donation for both fertile and infertile women is presented in Table-III. Most of the women in each group reported that they lacked sufficient enough knowledge about oocyte donation. Most of the women in both groups declared that they would not accept oocytes from another person if they did not or could not have a child. Similarly, most of the women in both groups declared negative answers about their husbands’ attitudes to oocyte donation. Most of the fertile and infertile women declared that they would not donate their oocytes to someone else who did not have a child. However, this proportion was significantly higher in fertile women. A significantly higher number of fertile women declared that they would accept an oocyte from one of their relatives. The same number of fertile and infertile women declared that they would not accept donating an oocyte to one of their relatives.

**Table-II:** Demographic data of the groups.

*Parameters*		*Fertile*	*Infertile*	p*
Age (years) [(Mean ± Standard deviation) (min-max)]		34.6±6.6 (19-44)	33.7±5.4 (21-44)	0.044
Education Level [n (%)]	Primary School	26 (19.5%)	16 (12.0%)	0.066
	High School	33 (24.8%)	35 (26.3%)	
	University	74 (55.6%)	82 (61.7%)	
Duration of marriage (years) [ (Mean ± Standard deviation) (min-max)]		6.0±3.3 (2-22)	6.5±3.6 (2-20)	0.241
Employment [n (%)]	Unemployed	41 (30.8%)	29 (21.8%)	0.095
	Employed	92 (69.2%)	104 (78.2%)	
Monthly income [n (%)]	Low	39 (29.3%)	19 (14.3%)	<0.001
	Normal	52 (39.1%)	41 (30.8%)	
	High	42 (31.6%)	73 (54.9%)	

n: number, Min-Max: minimum-maximum, SD: standard deviation

* Mann–Whitney U test.

**Table-III:** The distribution of answers to questions about oocyte donation for both fertile and infertile women.

*Question*	*Answers*	*Fertile Women*		*Infertile Women*		*p*
		*n*	*%*	*n*	*%*	
Do you have knowledge about oocyte donation?	Yes	16	12.0%	24	18.0%	0.170
	No	117	88.0%	109	82.0%	
Would you accept oocytes from donors if you had not or could not have a child?	Yes	16	12.0%	24	18.0%	0.111
	No	117	88.0%	109	82.0%	
Would your husband think of having a child from oocyte donation if you had not or could not have a child?	He would accept	10	7.6%	25	18.8%	<0.001
	He would not accept	97	72.8%	64	48.2%	
	I do not know	26	19.6%	44	33.0%	
Would you donate oocytes to someone else who wants to have a child from oocyte donation?	Yes	12	9.0%	25	18.8%	0.021
	No	121	91.0%	108	81.2%	
Would you accept oocytes from one of your close relatives?	Yes	29	21.8%	12	9.0%	0.004
	No	104	78.2%	121	91.0%	
Would you accept to donate your oocytes to one of your close relatives?	Yes	15	11.2%	15	11.2%	>0.999
	No	118	88.8%	118	88.8%	

## DISCUSSION

Having a child is an important goal in marriage and is seen as a vital means of stability and satisfaction in married life in many Islamic societies. The sociocultural burden of infertility is generally carried by the women, even when the detectable cause of infertility is associated with the male partner, as in the case of azospermia. Although reproductive medicine continues to help millions of couples to achieve pregnancy and relieves this social pressure, in some cases such as early menopause, congenital uterine anomalies, and azospermia with no sperm yield from testicular surgery, involvement of a third party is required in IVF procedures either by oocyte or sperm donation or by surrogacy.

Apart from cultural background, religion plays an important part in the decision-making and attitude of infertile couples and their families to gamete donation. Turkey is a secular democratic republic with a major Sunni Muslim population. Sunni Islamic clerics do not condemn the medical help to infertile couples as long as the lineage is protected. While IVF is a viable option to these couples, oocyte-sperm donation and surrogacy are all prohibited.

Our study showed that the vast majority of both fertile and infertile women had no knowledge of oocyte donation, though this study was conducted in a region of Turkey with a high education level. As a matter of fact, most of the participants were university graduates. We think that the lack of knowledge about oocyte donation originates from Turkey’s religious beliefs and the fact that oocyte donation is a prohibited practice in Turkey. However, a study performed by Baykal et al. 10 years ago showed that 25.1% of 368 married and infertile Turkish women had some knowledge about oocyte donation.^6^ This may be due to their study covering only infertile people and the more conservative trend of Turkish society in the last decades.

Isikoglu et al. conducted a public survey on 232 women in Antalya (Turkey) in 2005 concerning oocyte donation; they found that 29.74% of women had knowledge on oocyte donation.^7^ In that study, among 232 women, 160 were married and had children, and 72 women were single. Fifty percent of the women were university graduates. It is interesting that the oocyte donation, which is better known by infertile patients, is also known to some extent by the women with a child and single people. We think that this situation may be due to the cultural structure of Antalya and the fact that many people from Europe come to this city for health tourism.

In our study, 88% of fertile women and 82% of infertile women answered negatively about willingness to have children by oocyte donation. This shows that the Turkish community does not lean generally toward having children by means of oocyte donation; 91.0% of fertile women and 81.2% of infertile women answered ’no’ to the question “Would you like to donate your oocytes to someone else?” Neither fertile nor infertile women lean toward giving their oocytes to someone else. However, when groups were compared, the infertile women had a significantly more positive approach on the issue of “oocyte donation” compared to fertile cases. This suggests that the infertile women may be more sensitive to the problems of other infertile women.

Infertile women (91.0%) compared to fertile women (78.2%) had a more positive attitude to the issue of “oocyte acceptance from close relatives.” It is possible that the psychological effects of having no children can increase the infertile women’s rate of positive attitude on this issue. In addition, regarding oocyte donation to close relatives, both groups answered negatively in the same rate (88.8%). This result supports our prediction that the higher rate is seen in infertile women on the issue of “oocyte acceptance from close relatives” based on the troubled situation.

In the literature, there are also results that contradict the results that we have obtained in Turkish society. Akyuz et al. reported that 6 of every 10 infertile women will be able to donate their oocytes under special conditions (a close relative) and that more than half of them can accept the oocytes of somebody else.^8^ Baykal et al. reported that 23.3% of infertile women could accept oocyte donation from another woman and 33.8% of them could donate oocytes.^6^ Isikoglu et al. indicated in a study performed on fertile women that 82.76% of women had a positive attitude towards oocyte donation.^7^ In Sweden, Svanberg et al. stated that one out of every six women would donate their oocytes to someone that they do not know.^9^ Genuis et al. reported that 66% of participants in their study had positive attitudes to donating their oocytes to their siblings.^10^

We think that the country and region differences are influential on the results obtained in these studies. There are many differences between European countries and Turkey both in religious and cultural terms. The culture, the way of life, and the way of considering the events of each country and of the different regions within these countries can vary. The level of education, work status, stress factors, and marital status of the participants included in the studies vary.

## CONCLUSION

In conclusion, fertile and infertile women have no general knowledge on oocyte donation, and both fertile and infertile women lack a positive attitude to oocyte donation. We think that the negative attitude to oocyte donation is largely due to the cultural and religious structure of Turkish society.

### Authors’ contributions

**MMA:** Conception & design, acquisition of data, analysis, drafting the article, revision of the article, final approval.

**VU**: Analysis, revision of the article, final approval.

**KE:** Conception & design, acquisition of data, final approval.
